# Stress-Induced Volatile Emissions and Signalling in Inter-Plant Communication

**DOI:** 10.3390/plants11192566

**Published:** 2022-09-29

**Authors:** Joanah Midzi, David W. Jeffery, Ute Baumann, Suzy Rogiers, Stephen D. Tyerman, Vinay Pagay

**Affiliations:** 1 School of Agriculture, Food and Wine, The University of Adelaide, Glen Osmond, SA 5064, Australia; 2 Australian Research Council Training Centre for Innovative Wine Production, Urrbrae, SA 5064, Australia; 3 New South Wales Department of Primary Industries, Wollongbar, NSW 2477, Australia

**Keywords:** abiotic stress, biotic stress, plant–plant interactions, priming, stress signalling, VOCs, volatile-mediated signalling

## Abstract

The sessile plant has developed mechanisms to survive the “rough and tumble” of its natural surroundings, aided by its evolved innate immune system. Precise perception and rapid response to stress stimuli confer a fitness edge to the plant against its competitors, guaranteeing greater chances of survival and productivity. Plants can “eavesdrop” on volatile chemical cues from their stressed neighbours and have adapted to use these airborne signals to prepare for impending danger without having to experience the actual stress themselves. The role of volatile organic compounds (VOCs) in plant–plant communication has gained significant attention over the past decade, particularly with regard to the potential of VOCs to prime non-stressed plants for more robust defence responses to future stress challenges. The ecological relevance of such interactions under various environmental stresses has been much debated, and there is a nascent understanding of the mechanisms involved. This review discusses the significance of VOC-mediated inter-plant interactions under both biotic and abiotic stresses and highlights the potential to manipulate outcomes in agricultural systems for sustainable crop protection via enhanced defence. The need to integrate physiological, biochemical, and molecular approaches in understanding the underlying mechanisms and signalling pathways involved in volatile signalling is emphasised.

## 1. Introduction

Climate change has exacerbated the multifarious effects of environmental stresses on crop growth and development, thereby compromising sustainable agricultural productivity worldwide. Biotic stresses in the form of insects, bacteria, viruses, fungi, nematodes, arachnids, and weeds account for over 30% of losses from the annual global food production capacity, or approximately US$500 billion [1]. Abiotic stresses including drought, extreme temperatures, and salinity are major yield-limiting factors of economically important food crops globally [2]. Of these stresses, drought is one of the most significant, given that its frequency and severity has been forecasted to increase due to climate change [3]. Greater effort has therefore been directed towards the implementation of sustainable agricultural management and drought mitigation strategies in major crop-growing regions worldwide.

To compensate for their immobile nature, plants acclimatise to various environmental stresses with an array of complex molecular, physiological, and biochemical adaptations, which ultimately allow them to survive and even maintain potential rates of growth [4]. These adaptations may be expressed constitutively, or in many cases, are activated only in the presence of stressors. The evolved innate immunity or basal resistance of a plant is regulated by an intricate network of endogenous signalling molecules, receptor proteins, and transcriptional regulators. In response to environmental stress, plants exhibit an upregulation of the expression of stress-related genes encoding for defence proteins, such as trypsin protease inhibitors (PI) and pathogenesis-related (PR) proteins, which act against herbivory and pathogen attack, respectively. There is also an elevated production of secondary metabolites including osmoprotectants and toxins with deterrent/antifeedant activity [5,6,7,8,9,10,11,12,13]. Timely defence responses to biotic and abiotic stresses offer a fitness benefit to plants that largely depends on the capacity to quickly and accurately recognise external stress stimuli [14,15]. Upon stress perception, the plant will activate various defence-signalling pathways, leading to subsequent gene expression [15,16]. 

Among the secondary metabolites, phytochemicals in the form of volatile organic compounds (VOCs) have been identified as chemical-signalling molecules involved in both intra- and inter-plant communication, providing a fitness benefit to both the emitter and neighbouring receiver plants [17]. Plants respond to various biotic and abiotic stresses by emitting VOCs that fall into various compound classes, namely: terpenoids, benzenoids/phenylpropanoids, and fatty acid derivatives [17,18,19,20,21,22,23,24,25]. Recent studies in various crop species have explored an intriguing possibility of developing stress tolerance in plants through their exposure to VOCs from stressed neighbours, a process referred to as “priming” [5,6,7,8,9,10,11,12,13,26,27,28,29,30]. In the classical study by Karban et al. [27], volatile-mediated airborne signalling between native tobacco (*Nicotiana attenuata*) and sagebrush (*Artemisia tridentata*) was demonstrated. The sagebrush plants were clipped experimentally to mimic insect damage, and the emitted VOCs induced herbivore resistance in the neighbouring tobacco plants.

A follow-up study by Kessler et al. [31] reported an upregulation of herbivore-regulated genes in the receiver tobacco plants, but with no evidence of direct elicitation of defensive secondary metabolites. Interestingly, following post-challenge with *Manduca sexta* caterpillars, the receiver tobacco plants had an accelerated production of PI proteins, which was not evident in plants not previously exposed to the clipped sagebrush volatiles [31]. This study demonstrated how volatile-mediated plant–plant interaction primes defence responses in non-stressed receiver plants, inducing a faster and stronger response to a real stress. The production of defensive metabolites such as (*Z*)-3-hexenyl-vicianoside [12] and genes of defence-related enzymes including PI, threonine deaminase, and α-dioxygenase [31] have been shown to increase as a result of VOC exposure, ultimately conferring tolerance or resistance in non-stressed plants. Overall, however, the signalling pathways and mechanisms in volatile-mediated plant–plant interactions are vaguely understood and have been sparsely investigated in stress physiology studies. 

This review offers a comprehensive synopsis of volatile-mediated inter-plant communication, referencing studies published from 2000–2021 and using Google Scholar and Web of Science as the main academic search engines. It explores the range of VOCs that elicit various stress responses in non-stressed, neighbouring receiver plants in the face of both biotic and abiotic stresses. Understanding how plants can prepare for impending stresses by recognising VOC signals from stressed plants without themselves having to experience the actual stress is a noble endeavour towards the development of crops that are more tolerant against environmental stresses. Furthermore, the identification of specific receptors, transcription factors (TFs), and other regulatory proteins involved in volatile-mediated signalling, as well as the characterisation of the various defence-signalling pathways induced after VOC perception, is necessary for a full appreciation of airborne signalling between plants under stress. The integration of physiological, metabolome, and transcriptome analyses in volatile-mediated signalling and defence priming will be discussed.

## 2. Plant Volatile Organic Compounds

Plants emit and respond to a wide range of VOCs, which are generally lipophilic and of adequate volatility to be released into the atmosphere from the liquid phase [22]. Up to 10% of the carbon fixed during photosynthesis can be lost through complex volatile plumes [32]. Beyond terpenoids, which constitute the most complex group, other major classes of VOCs include fatty acid catabolites, aromatic compounds, and amino acid derivatives as products of the shikimic acid pathway [17,18,19,20,21,22,23,24]. Volatiles can either be emitted constitutively [33] or induced in response to factors associated with abiotic stress [21,22] or biotic stress [34]. VOCs have relatively high vapour pressure, low molecular weight, and low polarity properties, accounting for their high volatility [35]. The rate of emission from plant tissue into the atmosphere has been suggested to depend on the volatility, solubility, and diffusivity of the particular volatile compound rather than its rate of synthesis or other physiological mechanisms [36]. Nonetheless, the ratio of compounds in a constitutively emitted VOC bouquet is considered to be dependent on species taxonomy and varies greatly within species [37]. 

### VOC Biosynthesis

About 1700 VOCs have been characterised in different plant volatile blends, where they convey information about the plant identity and physiological condition [38]. The biosynthesis of VOCs occurs in the leaf mesophyll tissues, particularly in the palisade mesophyll cells, prior to their release either via inflicted wounds or the stomata on leaves and stems, which regulate emission rates by the opening and closing of their apertures [33,36,39]. The secretory cells of glandular trichomes are also active sites of VOC production [40]. In non-chlorophyll-containing tissues such as flowers and roots, VOC biosynthesis has been reported to occur in specialised crenulated epidermal cells, whose close proximity to the atmosphere or rhizosphere ensures immediate release [32,40]. Roots produce volatiles in epidermal cells and release them into the rhizosphere to mediate below-ground plant–plant interactions, thereby influencing the diversity of soil microbial communities [32,41,42].

**Figure 1:**
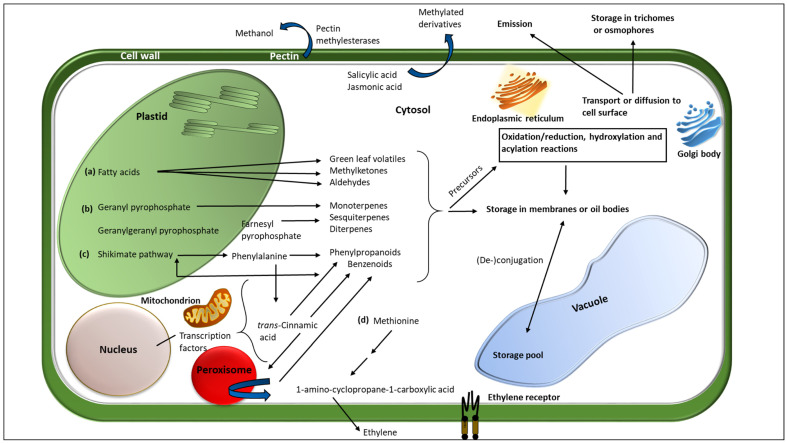
Biosynthetic sites, storage, and transport of major plant volatile organic compounds. The four major biosynthetic classes, (**a**) fatty acids, (**b**) terpenoids, (**c**) benzenoids, and (**d**) amino acids, are represented in the figure. Modification reactions including redox reactions, hydroxylation, acylation, and degradative reactions occur mainly in the cell cytosol. Some reactions may also occur in the membrane-bound sub-cellular compartments such as plastids, mitochondria, and endoplasmic reticulum. Inactive forms of the VOCs such as glycosides are stored in the vacuole, ducts, and other extracellular compartments such as trichomes. Transport of the volatiles is by passive diffusion and possibly via vesicular trafficking by the endoplasmic reticulum, golgi apparatus, and the *trans*-golgi network. (Modified from Pichersky et al. [51]).

Damage to the plant tissues triggers hydrolytic cleavage of complex membrane lipids by lipases to produce free polyunsaturated fatty acids such as the C_18_ linoleic and linolenic acids [43,44]. Following peroxidation of the fatty acids by lipoxygenase (LOX) enzymes, subsequent cleavage of lipid hydroperoxide by hydroperoxide lyase (HPL) will give rise to a suite of C_6_ aldehydes, alcohols and esters—collectively known as green leaf volatiles (GLVs) (Figure 1, plastid) [7,8,34,45]. In the presence of allene oxide synthase (AOS), the lipid hydroperoxide is rechannelled to the jasmonic acid (JA) pathway for JA synthesis [44]. Terpenoids form the largest and most diverse group of organic compounds synthesised via two distinct metabolic pathways: the cystolic mevalonic acid (MVA) pathway and the plastidic 2-C-methyl-D-erythritol 4-phosphate (MEP) pathway. All terpenoids are derived from C_5_ isoprene precursors, isopentenyl pyrophosphate (IPP), and dimethylallyl diphosphate (DMADP), with reactions catalysed by various terpene synthases [46] (Figure 1, plastid). Aromatic compounds (in terms of chemistry rather than any potential aroma) are derived from L-phenylalanine and include products from the chain-shortening of *trans*-cinnamic acid and structures derived from lignin biosynthesis that form benzenoids [19,47] (Figure 1, plastid). Additionally, amino acid methionine produces 1-aminocyclopropane-1-carboxylic acid (ACC), which is enzymatically oxidised to form ethylene [48] (Figure 1, cytosol). Methanol is also one of the most abundant VOCs found in plants and has been reported to serve as a signalling molecule in intra- and inter-plant communication [49,50]. It is produced through the methylation of the cell wall constituent pectin during plant growth and senescence. The high pH of herbivore salivary secretions is reported to also activate cell-wall pectin methylesterases, leading to copious amounts of methanol being released (Figure 1, cell wall) [40]. 

## 3. Plant Volatile Storage, Transport, and Emission

Considering that many of the VOCs produced by plants are likely to be lethal to the plant itself at high concentrations, several self-resistance mechanisms are employed by plants during VOC storage, transport, and emission to avoid self-toxicity (Figure 1). These include vacuolar sequestration, vesicle transport, extracellular excretion, extracellular biosynthesis, and storage of VOCs in cells as inactive non-volatile glycoside precursors [52,53]. Storage of VOCs occurs in various extracellular storage organs, including glandular trichomes, ducts, and laticifers, as well as the sub-cellular membrane-bound compartments such as vacuoles, plastids, mitochondria, and the endoplasmic reticulum (ER) [40,51,54]. 

Stored VOCs are released after deconjugation of their precursors upon mixing with lytic enzymes after the rupturing of storage organs through mechanical damage [40]. Studies have shown that specific components from herbivore salivary secretions such as β-glucosidases, glucose oxidases, and volicitin act as elicitors of HIPV emission during herbivory to release toxic aglycones of the glycoside conjugates, which have antibiotic and/or antixenotic effects on the herbivores [42,55,56,57,58,59]. When gut regurgitant of cabbage caterpillar *Pieris brassicae* was used in a study by Mattiacci et al. [60] to treat artificially damaged cabbages, a release of volatile blends similar to those observed from herbivore-damaged plants was noted. Parallel results were observed when β-glucosidase from bitter almonds was used to treat undamaged *P. lunatus* [61]. Interestingly, when lima bean plants were treated with JA solution, a similar VOC blend was emitted. It was postulated that the lytic enzymes in herbivore saliva hydrolyse cell structures into oligosaccharides and pectin, which in turn act as first signals leading to gene activation and de novo metabolite synthesis via internal signal transduction pathways such as the JA pathway [62,63]. 

Despite the presumption that VOC movement from the site of synthesis into the atmosphere occurs via passive diffusion, Widhalm et al. [32] questioned this supposition and highlighted the possibility of high barrier resistance in the movement of lipophilic VOCs across the cytosol, plasma membrane, aqueous cell wall, and cuticle. Movement via diffusion alone is likely to be too slow to account for the high emission rates observed during stress responses. Based on Fick’s first law of diffusion, it was determined that VOCs had to accumulate to toxic levels internally before they could be emitted at the observed emission rates, so it has been suggested that more active trafficking mechanisms may be involved [32]. Plausible mechanisms involve vesicular trafficking associated with the endoplasmic reticulum and Golgi apparatus [32,64], soluble carrier proteins [65], plasma-membrane localised transporters such as ATP-Binding Cassette (ABC) transporters [66,67], and small carrier proteins in the cell apoplast, such as lipid transfer proteins (LTPs) [68,69]. From these studies, it can be hypothesised that the same mechanisms that facilitate volatile emission from cells into the atmosphere may also be involved in VOC recognition and uptake [70].

Apart from VOC emissions from ruptured specialised storage structures such as glandular trichomes and ducts, emissions from plants are predominantly via stomata in leaves or directly from the epidermal cells in tissues lacking stomata, such as flower petals and roots. Emission of VOCs via the cuticle is an alternative pathway for some monoterpenoids. This has been calculated to contribute only 10–20% of the total monoterpenoid emissions, and therefore would not account for the high emission rates observed when stomatal conductance is low [71]. Stomata provide a low resistance pathway for volatile emissions, but they may decrease the efflux of VOCs from plants during stomatal closure resulting from various stressors [72,73]. 

The magnitude of stress-induced VOC emissions in a plant depends on its stress tolerance as well as the severity, timing, and duration of that particular stressor [21]. Unlike storage emissions, de novo emissions are generally more sensitive to stress and have been suggested to be the primary source of emitted isoprenoids [74,75]. Constitutive emissions can be altered through immediate stress responses and acclimation after stress recovery [76,77]. Volatiles including LOX-pathway products [78] and methanol [79] are induced during early stress (mild stress), reflecting signal activation at the membrane level and in cell walls [80]. Later stress responses include emissions of specialised isoprenoids [22]. Induced volatile phytohormones including MeJA, MeSA, and ET mediate stress signal perception, transduction, and propagation, leading to the activation of gene expression of defence-related genes, including enzymes involved in VOC synthesis [21]. The lifetime of these induced VOC emissions is finite following stress exposure. LOX products and MeSA have been shown to last for 15h after ozone exposure [79] and five to seven days under heat stress [81], and isoprenoid emissions continued for two days after herbivory [82].

Physicochemical and physiological factors limit VOC emission rates in plants, with the extent of control varying with the specific volatiles stored in the leaf [36]. Physicochemical constraints affect the emission of the synthesised VOCs to the ambient air by limiting their volatility and diffusion [36]. Leaf anatomy, specifically the presence of specialised VOC storage structures such as glands and resin ducts, has also been shown to influence emission rates [83]. Large diffusion resistances of various VOC storage pools exist between the intercellular spaces and the ambient air as a result of layers of epithelial and sclerenchyma cells lining the storage structures [74], unless rupturing of the pools occurs. Biochemical and molecular control over VOC synthesis in response to physiological factors such as light [84], temperature, drought [75,83], and high carbon dioxide concentration [83,85] has been shown to relate to the availability of immediate VOC precursors as well as on the activity rate of flux-controlling enzymes.

**Figure 2:**
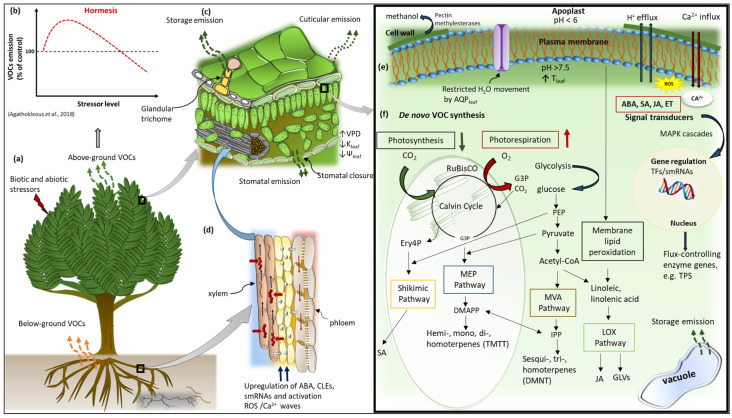
A simplified schematic of the interactions among major VOC biosynthesis pathways in response to stress. (**a**) VOC emissions respond to biotic and abiotic stresses from both below and aboveground sources of a plant. (**b**) A generalised VOC emission response to stress is given, which depicts a hormetic-like biphasic pattern. (**c**) Increased stress results in decreased intercellular CO_2_ concentration favouring photorespiration over photosynthesis, which is the source of VOC precursors. (**d**) Various root-to-shoot signalling molecules that are involved in stress signalling. (**e**) Membrane depolarisation activities involving ROS accumulation and Ca^2+^ influx as well as stress-induced phytohormones, e.g., ABA, JA, SA, and ET, activate defence signal transduction pathways that trigger gene expression of flux-controlling enzymes, e.g., TPS genes. Stressors affecting leaf temperature (T_leaf_) influence the activity of the enzymes. (**f**) De novo VOC synthesis in the chloroplast and cytosol involves VOC precursors such as G3P (for the MEP pathway) and Ery4P (for the shikimic pathway), PEP from glycolysis, as well as its downstream metabolites pyruvate and acetyl-CoA that are involved in the Shikimic, MVA, and LOX biosynthesis pathways. Note that the sizes of organelles are not drawn to scale. Abbreviations: ABA, abscisic acid; JA, jasmonic acid; SA—salicylic acid; ET, ethylene; TPS, terpene synthase; G3P, glyceraldehyde 3-phosphate; MEP, 2-C-methyl-d-erythritol 4-phosphate; Ery4P, erythrose 4-phosphate; PEP, phosphoenol pyruvate; MVA, mevalonic acid; LOX, lipoxygenase; CLE25, CLAVATA3/EMBRYO-SURROUNDING REGION-RELATED 25; smRNA, small RNAs [114].

The biosynthesis of signalling VOCs depends on the plastidic pool of intermediate products of photosynthesis, including glyceraldehyde 3-phosphate (G3P) and erythrose 4-phosphate (Ery4P), as well as sufficient supply of phosphoenol pyruvate (PEP) from glycolysis [74] (Figure 2). During mild stress, the photosynthetic machinery is affected such that the carbon assimilation rate is significantly decreased [86]. Emission rates of most VOCs are expected to be reduced under such conditions [84]. However, an increase in emissions after stress exposure has been observed during drought [76,87], heat [88], salinity [89], and ozone stress [90], indicating the acclimation of VOC synthesis under stress conditions and the plant’s ability to maintain high emission rates after stress relief. 

Mild water stress conditions, like most abiotic stressors, typically reduce stomatal conductance, thereby reducing intercellular CO_2_ concentration [86] and increasing leaf temperature because of constrained leaf transpiration [91]. The temperature response of VOC emissions is a function of increased enzyme activity and substrate availability [92], such that an increase in temperature reduces photosynthetic metabolites and energy, subsequently reducing de novo VOC synthesis and emission [93]. Although the reduction in stomatal conductance limits CO_2_ supply to the Calvin–Benson cycle of photosynthesis, photosynthetic electron transport is not inhibited, as the triggering of photorespiration may effectively supply CO_2_ and phosphoglycerate (G3P) needed to drive the cycle [21] (Figure 2). Therefore, ATP and NADPH remain available for the reduction in carbon reserves in the form of starch and sugar for isoprenoid synthesis [21]. Typically, sustained moderate or strong rapid drought stress will eventually lead to a significant reduction in VOC emissions, with augmented emissions after rewatering [76,87]. However, prolonged water stress can lead to accelerated leaf senescence and retardation in key terminal enzyme activity, leading to low emission rates during and after stress [84,94,95,96].

Mild heat stress enhances the activities of flux-controlling enzymes involved in the terpenoid synthesis pathway at the expense of carbon fixation enzymes. Subsequently, emission increases as the VOC synthesis pathway becomes more competitive for carbon and photosynthetic electrons than carbon fixation [88,94]. Increases in temperature also affect photorespiration, which can indirectly regulate PEP levels available to form intermediate substrates for VOC biosynthesis [97]. Light intensity affects the amount of the terpenoid precursor G3P available from photosynthesis, as well as the energetic co-factors ATP and NADPH required for its chemical reduction [98].

The diffusion of individual VOCs is influenced by the width of the stomatal opening, the leaf anatomy, and compound molar size [36]. The volatility of a specific VOC is determined by equilibrium partitioning between the intercellular airspaces and the ambient air, which then affect the diffusion gradient [36,74]. Changes in stomatal conductance can differentially affect the emission rates of various VOCs under stress conditions [39]. The emission of methanol [99], linalool [39], acetic acid [100], and acetaldehyde [101] were vulnerable to stomatal conductance, whereas several monoterpenoids such as limonene, *trans*-β-ocimene [39], isoprene [102], and α-pinene [103] were emitted independently of stomatal regulation. This independence from stomatal conductance has been associated with the large Henry’s Law constant (*H;* gas/liquid phase partition coefficient) of the monoterpenoids, which implies that they can maintain a high intercellular partial pressure for a given liquid phase concentration. An increase in the diffusion gradient from the intercellular space to the external atmosphere may compensate, at least partially, for the reduced stomatal conductance, in order to control VOC emission rates [39,102,104]. 

Following an earlier hypothesis that VOC emissions in plants may display hormetic responses to environmental stressors [105], studies involving herbivory [106,107], drought [108,109], CO_2_ concentration, light, temperature [92,110], and ozone [105,111,112] have also provided evidence that VOC emission is biphasic by time and dose in response to the stressors, thus indicating hormesis [113,114]. Hormesis is a biphasic stress dose response that depicts adaptive responses of organisms to low-dose stresses whereby they can improve their tolerance to severe stress challenges, whereas higher doses of the stresses will have negative effects on organisms [115] (Figure 2). The occurrence of hormesis of VOC emission in plants suggests an evolutionary adaptation that acts to maintain fitness in a changing environment in the context of enhancing intra- and interplant communication, defence priming, protection, and defence functions [114].

## 4. Volatile Uptake, Perception, and Signalling

The adsorption of VOCs on the surface of the leaves as well as their solubilisation and enzymatic detoxification within the leaves are what drive the uptake, deposition, and storage of VOCs [116]. VOCs’ chemical reactivity, physicochemical properties, and involvement in leaf metabolism all play a role in how much they react in leaves and can potentially be absorbed by vegetation [116]. Though stomatal uptake is a significant sink for VOCs in leaves, VOCs can adsorb directly onto the cuticle as a result of gas-phase deposition [117,118,119,120]. The cuticle opens up as a route to and from the leaf tissues for organic compounds with substantial lipophilicity and low vapour pressure in addition to the stomatal route [118]. The degree of stomatal conductance along with the combination of VOC physicochemical properties will determine whether VOCs are released or absorbed through the cuticle, the stomatal pores, or both.

The direction of the concentration gradient between the leaf intercellular air space and the surrounding air determines whether a certain VOC is released or absorbed at any particular time. At very low internal concentrations, the uptake rate is controlled essentially by stomatal conductance as well as the ambient concentration of the VOC [116]. A more significant uptake of monoterpenes was observed when the ambient air concentration was higher versus when it was lower [121]. The thickness and conductivity of the leaf boundary layer also have a bearing on VOC foliar uptake and deposition, which itself is influenced by leaf morphology and wind speed [119]. Higher uptake rates have been noted in plants with thinner leaves and greater surface area per unit dry mass [121]. Overall, it is anticipated that temperature, VOC and cuticle physicochemical properties, and cuticular structure would all play major roles in regulating the deposition and release of compounds from leaf surfaces [116].

Once deposited on the plant’s surface, stress-induced VOCs may act as resistance elicitors perceivable by the plant’s evolved and highly sophisticated surveillance system. Subsequently, unique molecular mechanisms of several defence-signalling pathways are initiated [122,123], thereby conferring some degree of immunity in distal regions of the same plant or a neighbouring plant, thus acting as long-distance damage-associated molecular patterns (DAMPs) [124,125]. Elicitation by the volatile DAMPs ranges from early signalling cascades (including Ca^2+^ influxes, oxidative burst, and MAPK activation) to phenotypic defence responses [126]. Methanol emissions in plants, just like GLVs, are reliably associated with injury and have been suggested to act as wound signals in plants [49]. Studies have shown that methanol activated oxidative burst and MAPK cascades in *Festuca arundinacea* (tall fescue) grass and tomato [127]. Isoprene was shown in *Arabidopsis* to induce signal transduction networks associated with stress response and plant growth regulators, such as JA, SA, and ET, which promote defence [128,129].

Although the molecular basis for VOC perception and signalling is vaguely understood, it is likely that VOC signals are first perceived at the membranes, making the transmembrane pathway a more plausible route for signal generation and transduction. On the other hand, a plant’s response could be simply evoked by volatiles that might be deposited on the plasma membrane due to their lipophilic or amphiphilic nature, which could probably distort the membrane organisation [130]. Subsequent VOC metabolisation or VOC recognition in the cytoplasm of those VOCs that manage to pass through the membrane is also a possibility, as observed in studies related to the uptake of ^13^C- labelled GLVs in plants [131,132]. Unlike most VOCs, ethylene perception and signal transduction have been well-established through extensive studies using *Arabidopsis* [133,134,135,136,137,138,139,140]. First suggested by Burg and Burg [133], this gaseous phytohormone binds reversibly to its receptor via a protein-bound transition metal co-factor such as copper. Compelling evidence from biochemical data has shown that the ethylene signal is perceived at the membrane of the endoplasmic reticulum by a family of receptors, including ETHYLENE RESPONSE1 (ETR1), ETHYLENE RESPONSE SENSOR (ERS1), ETR2, ETHYLENE INSENSITIVE 4 (EIN4), and (ERS2), which repress ethylene through the negative regulator CONSTITUTIVE RESPONSE1 (CTR1). Downstream and secondary ethylene responsive gene expression are positively regulated by EIN2 and EIN3 [137,139,141,142,143]. 

Efforts to understand the molecular aspects of VOC perception and signalling have involved studies in exogenous applications of VOCs to plants. In these studies, VOC-binding proteins involved in gene transcriptional regulation have been identified [70]. A transcriptional co-repressor, TOPLESS-like protein (TPL), was shown in tobacco to bind to β-caryophyllene, a sesquiterpene, to initiate the expression of stress response gene *NtOsmotin*, a pathogenesis-related protein [70]. The study suggested significant specificity of the receptors for individual VOCs as well as selectivity in inducing gene expression in plants. Though the involvement of membrane receptors in the VOC-sensing mechanism cannot be ruled out, it is clear that some nuclear proteins may act as VOC receptors in plants. 

Documented studies on mechanisms by which plants perceive various stimuli such as light [144,145], sound [146,147,148,149], and touch [150] have shown uncanny similarities with those exhibited in mammalian systems. It is also probable that the mechanisms involved in how plants “smell” odours follow the same paradigm as that observed in mammals—excluding the existence of a nervous system. In mammals, the olfactory system has been described as discriminatory, in that each volatile compound is perceived to have a distinct odour, and even subtle changes in odourant structure or concentration can potentially alter the VOC’s “code”, thereby shifting the perception of its odour quality [151]. A similar discriminatory behaviour has also been observed in the parasitic dodder plant (*C. pentagona)*, which locates its host plants based on its perception of their unique volatile blends [152] and is repelled by specific VOC blends of its non-hosts. In mammals, a combinatorial receptor coding scheme is used by the olfactory system, where specific combinations of odourant receptors (ORs) recognise specific volatiles. Studies by Malnic et al. [151] have shown that even subtle alterations in the volatile compound or changes in its concentration may result in changes in its “code”, thereby affecting the perceived quality of the odour. Whether plants follow a similar model for their VOC receptors remains unclear, but it is worth considering in future endeavours to understand the mechanisms of volatile perception and signalling.

## 5. Volatile-Mediated Intra- and Inter-Plant Communication

**Figure 3:**
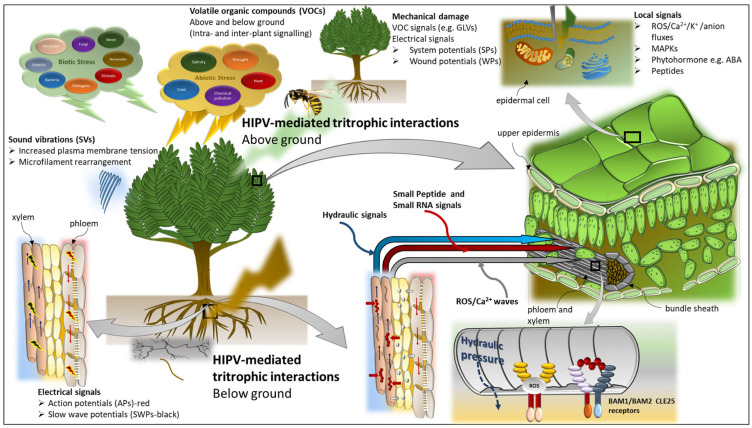
Overview of long distance signals in plants. Long-distance signals include electrical, hydraulic, and chemical signals. Electrical signals found in plants include: slow wave potentials (SWPs) propagated in the functional xylem; action potentials (APs) initiated in the phloem; system potentials (SPs) propagated in the apoplast following mechanical perturbations or wounding; and wound potentials (WPs) through changes in cell turgor leading to plasma membrane depolarisation. Hydraulic signals involve changes in turgor pressure, mass flow, and pressure waves. SWPs are closely linked to hydraulic signals as a result of cavitation events or changes in turgor [154,155,156]. Chemical signals can be classified as: (i) secondary messengers including reactive oxygen species (ROS), inositol triphosphate (IP), Ca^2+^, K^+^, and anion fluxes; (ii) signalling cascade chemicals including mitogen-activated protein kinases (MAPKs); and (iii) chemical response signals including phytohormones and volatile organic compounds (VOCs). Herbivore-induced plant volatiles (HIPVs) have been well-documented involving both above- and below-ground biocontrol of herbivores by insect predators and parasitoids, such as the yellow jacket wasp and the parasitic wasp, respectively [157,158]. Recent advanced analyses have elucidated the role of various mobile molecules including small peptides [155,159,160] and small RNAs (small interfering RNA and micro RNAs) in long-distance systemic signalling [159,161,162,163]. Stomatal closure is one of the initial responses to osmotic stress to prevent hydraulic failure [164] and is regulated by abscisic acid (ABA) [165,166]. During water stress, the root-to-shoot communication is mediated by a small mobile root-derived peptide, CLAVATA3/EMBRYO-SURROUNDING REGION-RELATED 25 (CLE25), which then triggers ABA accumulation in the leaves via BARELY ANY MERISTEM1 (BAM1) and BAM2 receptors in the leaf vascular bundles [159]. Sound vibrations (SVs) or acoustic signals produced by both biotic and abiotic stresses act as stimuli capable of priming plants for future stress challenges and as long-range signals that activate plant-signalling pathways [148]. Leaf vibrations caused by herbivore chewing [148,149] and the “clicking” sound produced by the collapsing water column (cavitation) in the xylem [167] have been demonstrated to trigger systemic responses in distal regions of the plant. The perturbation of the plasma membrane by SVs is characterised by a sequence of molecular episodes including cell wall modification and microfilament rearrangement in plant cells [147,149,168].

Plants have evolved unique communication mechanisms between various organs for the enhancement of their development and providing resistance to stress. Upon perception of an environmental stress, a plant will activate several long-distance signals to trigger systemic stress responses with the aid of the plant’s vasculature (Figure 3). Environmental information can be conveyed from the roots to the aerial parts of the plant, and vice-versa, via well-orchestrated cell-to-cell and/or long-distance signals. This root-to-shoot/shoot-to-root signalling is crucial in the activation of defence responses in the target tissues, which allow for plant adaptation to both biotic and abiotic stresses at the whole-plant level. These possibly interconnected signals, which include electrical, hydraulic, and chemical signals, differ in their mode of action as well as propagation speed [153,154]. As a consequence of multiple stresses, these signals tend to overlap in their presence and speed, making it difficult to associate a particular response to a specific stress stimulus [154,155].

Unlike most chemical signals, VOCs are long-distance signals that can induce systemic stress responses in distant plant organs that have little or no distinct vascular connection with parts of the stressed plant [169,170]. The role of volatiles in intra- and inter-specific communication, their chemical diversity, and mode of action is well-documented [20,171,172,173,174,175,176,177,178,179]. They also serve as signalling molecules that are involved in intra- and inter-plant communication, providing a fitness benefit to the “emitter” and modifying behaviour in neighbouring “receiver” plants [17]. 

Plants are reported to be able to distinguish between the different volatile blends [152]. As individual VOCs are not species-specific, plants formulate unique messages by adjusting the individual components of a volatile bouquet [180], which in turn induce specific responses in the receiver. For instance, the parasitic dodder plant (*Cuscuta pentagonia*) uses specific VOC blends as foraging cues to locate its host plants [152]. The presence of (*Z*)-3-hexenyl acetate in the constitutive VOC blend of wheat, a non-host to the dodder plant, was reported to be absent in tomato, suggesting its role as a repellent to the dodder [152].

Volatile blend composition has a bearing on the specificity of volatile-mediated plant–plant communication in congeneric species such that “eavesdropping” by other species can be avoided [27,180,181]. In such cases, competing plant heterospecies may not be able to “decode” the chemical stress signals, which may otherwise have served to prepare them for a potential threat [179,182].

The allelopathic effect of VOCs, particularly terpenoids, has also been employed as part of the plant’s defence against its competing neighbours by remotely suppressing the competitors’ growth [183]. The cellular mode of action that underlies the allelopathic effect of terpenoids, such as eucalyptol, β-myrcene, camphor, camphene, menthol, and α- and β-pinene, occurs via cytoskeletal disruption, with microtubules being the primary targets [184,185,186,187,188]. Essential oils containing these terpenoids have been used for weed control as well as biological control of pests [188,189], facilitated by the specific nature of allelopathic compounds [190,191,192].

Under biotic stress, plants respond to insect and pathogen attacks by emitting herbivore-induced plant volatiles (HIPVs) and pathogen-induced volatiles (PIPVs), respectively [30,193,194,195,196,197,198]. Such volatiles may be exploited by neighbouring hetero- or con-specifics to either benefit or compromise the fitness of the emitter plant. The HIPVs/PIPVs can either benefit the emitter plant by attracting pollinators and natural enemies of its herbivores, or they can negatively affect the plant by acting as foraging cues to parasitic plants and as host location cues by herbivores [40,152]. Interestingly, in some HIPV-mediated plant interactions, receiver plants have been shown to adsorb VOCs from their stressed neighbours and process them into their lethal derivatives, which are detrimental to herbivore growth and survival [12]. In some instances, the receiver plant will re-release VOCs that have been adsorbed from the source plant against insect pests, in what is known as associational resistance (AR) [199]. 

**Figure 4:**
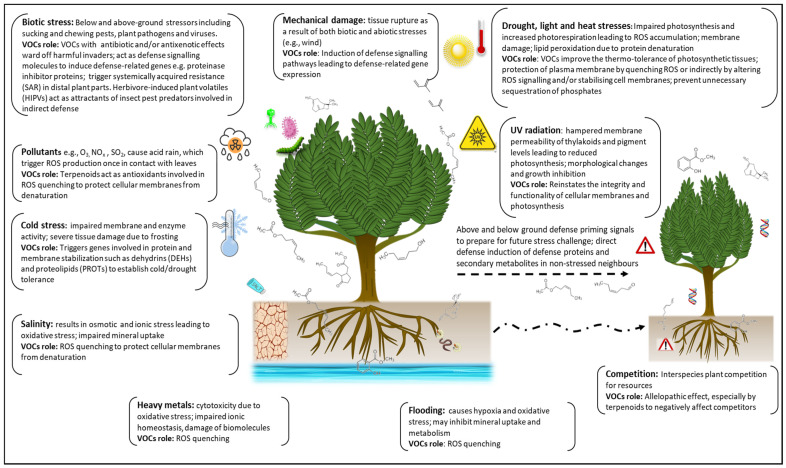
Role and mechanism of action of stress-induced volatile organic compounds (VOCs). In response to both biotic and abiotic stresses, VOCs induce various defence-signalling pathways and are involved in intra- and inter-plant priming against future stress challenges. The antixenotic and antibiotic effects of VOCs facilitate direct and indirect defence responses against biotic stressors. Both abiotic and biotic stress induce the production of reactive oxygen species (ROS) whose negative oxidative effects are curbed by VOCs through ROS quenching and cell membrane stabilisation. Thermo-tolerance of photosynthetic tissue is also facilitated by some VOCs under high temperature conditions. (Modified from Vickers et al. [208]).

Abiotic factors such as high light intensity [200], nutrient availability [201], salinity [11], temperature [29,72,73,104,202], wind and UV radiation [203,204], ozone exposure [205], and mechanical damage [5] have also been shown to induce VOC emissions in plants. In response to abiotic stress, VOCs offer protection against high-temperature exposure in addition to improving the thermo-tolerance of photosynthetic tissues [22,206,207,208,209,210]. To alleviate the negative effects of oxidative stress induced in response to environmental stressors, plant VOCs can stabilise and protect cellular membranes by quenching ROS species or by altering ROS signalling [211,212]. The role and mechanism of action of stress-induced VOCs in curbing the detrimental effects of biotic and abiotic stresses is summarised in Figure 4. 

The potential role of stress-induced VOCs in priming neighbouring plants for enhanced stress tolerance has been explored [5,6,7,8,9,10,11,12,13,27,29,30,213], with only a few studies focusing on abiotic stress. Priming is an adaptive strategy characterised by an enhanced responsiveness of defence mechanisms to stress as a result of a previous stress challenges. This involves subtle physiological, molecular, and epigenetic alterations in the plant leading to increased stress resistance and/or tolerance [214]. GLVs comprise an important chemical group in the HIPV/PIPV plume emitted in response to mechanical damage, herbivory, or pathogen attack. The priming effect of GLVs has been characterised by an increase in defence-related gene expression and an augmented production of secondary metabolites with antixenotic or antibiotic effects on the biotic stressors [20,193,215,216]. In studies that involved priming for salinity stress, a significant increase in salt tolerance was observed in *Arabidopsis* and lima beans (*Phaseolus lunatus*) plants, independent of ABA and salinity stress-signalling pathways) [11,13]. An increase in photosynthetic rate and relative growth rate was observed in the plants previously exposed to VOCs from salt-stressed plants. In a similar study by Landi et al. [28], enhanced photosynthetic activity and reproductive success was also observed in *Ocimum basilicum*. In this particular study, the observed VOC profile differences of the emitter and the receiver plants were thought to suggest the ability of the receiver plants to propagate the volatile signal to their own neighbours [28]. Beyond these examples, Table 1 gives a brief account of a selection of studies on VOC-mediated priming in plant–plant communication under biotic and abiotic stresses.

Apart from the GLVs that are released in response to herbivory and mechanical damage, terpenoids including monoterpenes, sesquiterpenes, and hemiterpenes have also been identified and associated with defence priming in receiver plants. Typical VOCs include monoterpenes β-ocimene, 1,8-cineole, linalool, α- and β-pinene; sesquiterpenes β-caryophyllene, (*E*)-α-bergamotene and (*E*)*-*β-farnesene; hemiterpenes *(E*)-4,8-dimethylnona-1,3,7-triene (DMNT), and 4,8,12-trimethyltrideca-1,3,7,11-tetraene (TMTT). Phenylpropenes including eugenol and methyl eugenol were detected in salt-stressed plant emissions.

## 6. Priming: The Cost of Defence

The concept of priming, an indication of hormesis, has gained interest in plant stress physiology due to its feasibility and efficiency, as well as being a cost- and resource-effective tool [8,214]. Priming has been associated with induced resistance (IR) whereby a plant is sensitised for a more robust basal defence response to subsequent attacks [217]. This immunological memory can last throughout the life cycle of a plant and is possibly heritable [214]. IR thus puts the plant in an alert state for future stress challenges and confers broad-spectrum protection against various environmental stresses [214]. 

To ascertain that effective defence priming has been achieved, more robust defence responses with a low fitness cost must be observed. In addition, a superior response in the presence of the actual stress challenge must be evident to imply that the plant has a memory of the stress stimulus [218]. In the context of priming using VOCs, a receiver plant must be able to associate a particular volatile plume to a specific stress in order to affect corresponding and specific defence responses. The priming of defence responses in receiver plants due to volatile signals may depend on the plant’s receptiveness to different concentrations of specific volatile blends or individual compounds, the duration of exposure, and the effective distance from the source plant before the VOC bouquet is diluted in the air to inactive concentrations [42,180,219,220]. In the latter case, as the VOCs are released into the atmosphere, eddy currents dilute them to concentrations that are adequate to prime neighbouring plants for impending stresses [217,221,222,223]. Since the concentrations of airborne VOC signals required to induce priming are generally lower than those in which a full defence response is elicited [223], low fitness costs are likely to be incurred by the emitter plants. Although it has been presumed that priming does not alter plant metabolic pathways or initiate defence-related gene expression until actual exposure to the stress [11,218,221,224,225], a study by Balmer et al. [226] has shown that direct changes for enhanced tolerance can in fact be triggered with negligible fitness costs in the receiver plants. Priming may, therefore, offer an ecological fitness benefit to plants that are able to launch faster and stronger defence responses when the impending stress arrives [31,227]. 

Besides the ability to prime or induce defence-related responses in receiver plants, VOC exposure may have the potential to affect nutrient uptake and photosynthesis, thereby modulating primary and secondary metabolisms. This would subsequently lead to a change in the overall growth and development of the plants [228], as observed in VOC-exposed broad bean plants that reduced their photosynthetic rates in response to VOCs whilst they achieved increased salt resilience [13]. A reduction in photosynthesis was also shown in sweet basil plants only after subsequent exposure to salt stress, and they were observed to have early flowering, a greater seed set, and early senescence [28]. A downside of priming has also been observed in studies reporting the downregulation of specific resistance pathways, potentially leading to a reduced defence ability to unrelated stressors that the plant may not have been primed for [214].

This has been observed in transgenerational priming, in which inherited defence hormone crosstalk seemed to compromise the primed progeny’s defence response against stresses the parents had not been primed for [214]. Using the study by Luna et al. [229] as an example, it was observed that JA-dependent defence responses were compromised in salicylic acid (SA)-primed *Arabidopsis* progeny, leading to significant susceptibility to necrotrophic fungi [229]. This was accounted for by the probable existence of crosstalk between the JA and SA pathways.

Continued metabolic investment in the primed state in the absence of stress has a low fitness cost to a plant, but it may compromise growth and yield [214,230]. In a study carried out by Crisp et al. [230], the growth and yield of *Arabidopsis* plants previously primed by exposure to drought or high light intensity was significantly reduced in one group of plants, whereas another was observed to recover to their pre-stressed state after rewatering. The latter group was deemed to have “forgotten” about the primed state in contrast to the former, which maintained the hardened/primed state whereby defence responses remained activated. Considering that stresses are usually transitory and often repetitive in a given environment, plants that are able to balance the formation of stress memories for protection against future stress challenges with resetting for normal growth and yield may have developed an evolutionary adaptation strategy that allows them to compete effectively against their con- and hetero-specifics [230]. Where priming is observed to be maladaptive in transgenerational inheritance, plants would be better off resetting their memory and “forgetting” previous stresses to avoid compromising their growth and survival [230].

Overall, priming offers an opportunity for the development and implementation of sustainable crop protection strategies for Smart Agriculture [231]. Whereas studies on volatile-mediated priming of defence responses against biotic stresses in plants have been well-documented [5,6,8,27,31,169,172,222,232], they exist only sparsely for abiotic stresses [11,13,233]. Therefore, the need to explore opportunities that VOC-mediated priming could offer against the various abiotic stressors is greatly emphasised.

## 7. Exploiting VOC-Mediated Signalling for Future Sustainable Agriculture

### 7.1. The Ecological Aspects: A Community Perspective

In nature, a plant may respond to a myriad of signals from its neighbours—above and below ground—and/or under different environmental conditions [234]. Despite their sessile nature, plants are able to modulate their phenotype in response to a plethora of environmental stresses and to interactions with various members of their associated community. In these communities, plants are unlikely to experience single stress factors sequentially but rather multiple stresses simultaneously [157,234] while also interacting with both con- and hetero-species [235]. The co-occurrence of multiple stresses may have some additive effects, although in some cases, only one stress takes precedence. This means that plant response due to several stress combinations cannot be accurately extrapolated based on responses to individual stress factors [236], suggesting an interaction effect of the combined stresses on plant response. This too will result in a diverse mix of VOCs being emitted by the plant. In the context of VOC-mediated interactions, the cocktail of stress-induced VOCs emitted by the individual plants contributes significantly towards the complex transcriptional responses observed in the neighbouring plants [237]. It is plausible that receiver plants acquire a stronger basal resistance resulting from the stress-specific VOC blend, rendering them more resilient and fit in the face of diverse future stressors as they remain exposed to the different volatile blends over time. 

Studies on volatile-mediated plant–plant communication have indicated its ecological and functional relevance, although most work has understandably been carried out in controlled environments such as glasshouses and growth chambers due to the complexities of building a robust experimental design as well as incorporating adequate controls [238]. Evidence from studies on the volatile-mediated priming of non-stressed plants under various environmental conditions inspires the exploration of further opportunities to induce tolerance in crops against a broad spectrum of stresses. However, this should include moving on from studies carried out under controlled environmental conditions to those that better represent that which occurs in the natural environment. Normal agricultural management activities such as pruning, hedging, and crop harvesting, as well as key phenological events such as flowering and fruit set, are also potential factors that induce VOC emission in plants. The questions of how the neighbouring con- and hetero-specifics respond to the resultant volatile bouquet released by the multi-stressed plant and whether they will be primed for broader tolerance require exploration. Additional considerations include the examination of the effect of wind eddies on the airborne signals, because these may dilute the volatile bouquet to concentrations that may not effectively prime the intended receiver plants as mentioned earlier; the effective distance between the source and receiver plants in different agricultural systems; and diurnal changes in humidity and temperature, which will impact the volatility of the VOCs. These considerations will be relevant in ascertaining the feasibility of plant–plant VOC priming for enhanced stress resilience.

### 7.2. Technologies for Exploring Plant VOC Signalling Interactions

Attempting to address both the mechanistic and functional-level questions concerning inter-plant VOC signalling, several advances in the fields of metabolomics, volatilomics, transcriptomics, and bioinformatics offer an opportunity for a more holistic understanding of VOC-induced defence responses in plants. Significant progress in the development of VOC collection and analysis technologies that are sensitive and relatively inexpensive has been observed over the past decade [239,240,241]. These advances include gas chromatography-mass spectrometry (GC-MS) and static and dynamic headspace (SHS, DHS) techniques.

The headspace VOC sampling methods involve non-solvent sorptive extraction techniques, which include headspace solid phase microextraction (HS-SPME) and purge-and-trap headspace (P&T-HS) for static and dynamic extraction, respectively [239,242,243], using collection chambers (refer to Figure 4 in the review by Tholl et al. [239]). Live plants can be analysed with such techniques to provide a more representative volatile profile than with traditional methods such as solvent extraction or steam distillation. It is important, however, to consider changes that may occur in the plant’s micro-environment while in the headspace collection chambers. Increases in humidity [39] and temperature, as well as reduced light intensity in the chambers [244,245] are likely to affect transpiration rates and, subsequently, VOC composition and emission rates. Additionally, given the potential for the scalping of VOCs by hydrophobic materials, their adsorption by the lining of collection chambers or sampling bags may require the use of non-reactive materials such as fluorinated ethylene propylene (FEP) or polytetrafluoroethylene (PTFE), which have been shown to be effective in plant VOC sampling [210,246,247].

Innovations that allow for VOC sampling under natural environments in which plants grow have emerged in the past decade. Trends include direct analysis in real time (DART), which is an ionisation technology for rapid non-contact analyte detection on solid or liquid surfaces [248]. Portable devices suitable for both laboratory and field sampling include a portable gas chromatograph (GC) with photoionisation detector (GC-PID) such as the FROG-4000 VOC analyser. Volatile analysis using these devices can be undertaken in water, soil, and air [249]. Micro-versions of these detectors, μGC–PID, are currently being developed and have been demonstrated to facilitate rapid vapour analysis in the field [250]. The FlavourSpec is another portable analytical device consisting of GC coupled to an ion mobility spectrometer (IMS), which can analyse headspace volatiles of solid and liquid samples without prior treatment [243,251]. Additionally, another very cost-effective field sampling technique involving minimum headspace and organism manipulation involves the use of polydimethylsiloxane (PDMS) in the form of silicone tubing. This technique involves using small pieces of the tubing for headspace sampling, combined with thermal desorption (TD)-GC-MS analysis [252]. GC-independent methods such as proton-transfer reaction-mass spectrometry (PTR-MS) have also been relevant in both field and laboratory real-time VOC monitoring [253]. A high-resolution version of the PTR-MS uses a time-of-flight mass spectrometer (PTR-TOF-MS) that is capable of rapid measurement of VOCs at ultra-low concentrations with a resolution of isobaric ions [254]. A brief highlight of the various practical and novel technologies being employed in the metabolite profiling of VOCs is given in this review (Table 2); however, more detailed and extensive reviews have been documented.

With the advent of high-performance computing and statistical analysis packages such as R, there is an opportunity to mine and analyse the data collected from one or a combination of the above-mentioned methodologies to unveil relationships between VOC signals and plant physiology that would allow for a systems-based understanding of plant communication. Furthermore, data fusion approaches [255] that combine real-time VOC signalling measurements, e.g., PTR-MS, with high temporal resolution phenotyping data such as plant growth, spectral responses, and water and nutrient status are available. Modern “omics” methods such as metabolomics, volatilomics, proteomics, and transcriptomics methods all generate vast quantities of data requiring advanced analytics such as chemometrics, machine learning, and deep learning techniques for statistical analyses and prediction of plant responses to biotic and abiotic stresses. For example, Principal Component Analysis (PCA) is a multivariate statistical method to group variables and highlight their relative contributions to other variables based on variance. In contrast, machine learning algorithms based on supervised learning are available, which provide extensive and reliable datasets and can offer significant advantages over traditional chemometrics methods with regards to the prediction of VOC responses,; Random Forest is one example [256,257]. 

Though the use of physiological data in the evaluation of VOC signalling is relevant under both biotic and abiotic stresses, the impact of the signalling may be overlooked or assumed to be absent if the parameters selected in the study are influenced by several other environmental factors. For instance, in trying to investigate VOC signalling under abiotic stresses such as drought, it may be reasonable to consider observing changes in transpiration rate in the receiver plant. However, transpiration rate may not give a clear indication of signalling due to factors such as glasshouse lighting and stomatal oscillations [258], which depend on diurnal variations. Whether or not the receiver plant is responding to VOCs or to its inherent circadian rhythm may not be well-defined. Other physiological parameters such as ROS scavenging, stimulated root growth, and xylem hydraulic changes may be more difficult to measure directly. A more reliable option may be to investigate the subtle changes that could occur at the transcript level, which would offer an opportunity to understand the immediate responses of the receiver plants to VOC perception. Targeted gene expression using quantitative PCR (qPCR) is relevant in this regard and has been employed in several VOC-signalling studies under biotic [31,259] and abiotic stresses [11]. However, only a few putative genes can be investigated at a time, and those may not represent all the genes involved in VOC signalling. Investigating the gene expression of rate-limiting genes involved in GLV and terpenoid biosynthesis, as well as the various phytohormone signalling pathways, may be important for understanding VOC signalling in plants.

As opposed to qPCR analysis, which determines gene expression levels of a few pre-selected gene transcripts, next generation sequencing (NGS) technologies such as RNA-seq provide in-depth knowledge of a myriad of genes involved in various metabolic pathways and the complex interactions of plants during stress [260]. Gene expression profiling and transcriptome analyses are functional genomics approaches that have facilitated the identification of two broad classes of genes associated with stress responses. One category encompasses genes encoding the proteins involved with cellular osmotic homeostasis and stress protection, and the other consists of TFs and kinases involved in the regulation of stress transduction and gene expression in conjunction with various phytohormones [261]. NGS reveals transcript information, allowing for the identification of unique genes and single nucleotide variants, as well as allele-specific gene expression [261]. The platform offers an opportunity to investigate possible VOC protein receptors, TFs, and regulatory proteins involved in volatile signalling under various environmental stresses, which could prove invaluable in future studies. 

To complement gene expression studies, the use of genetically modified plants possessing the ability to either emit or perceive specific components of the wild-type volatile bouquet will be useful in understanding plant–plant VOC signalling [222]. The use of mutant “deaf” receiver plants with functionally impaired VOC receptors for specific volatile substances [141] and “mute” emitter lines with silenced genes of enzymes needed for VOC synthesis [262,263,264,265,266,267] may be useful in investigating the possible receptors involved in VOC signalling. Confirmation studies involving the use of synthetic analogues of the VOCs could then be undertaken in conjunction with the “mute” emitter plant to ascertain whether the response by receiver is restored after a specific VOC has been made absent in the “mute” emitter. 

**Figure 5:**
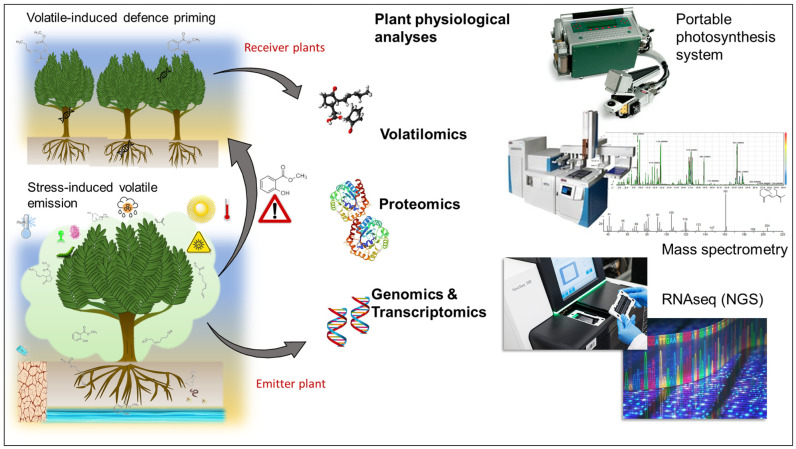
Integrating physiological, metabolome, proteome, and transcriptome analyses in understanding plant volatile signalling.

Efforts directed towards understanding VOC signalling pathways and defence priming can greatly benefit from the integration of physiological, metabolome, proteome, and transcriptome analyses (Figure 5), aided by the technological advancements in those individual fields witnessed over the past decade.

## 8. Conclusions and Future Perspectives

The increasing burden of climate change has exacerbated the effects of both biotic and abiotic stresses, thus posing a threat to global agricultural production. The employment of VOCs to enhance plant resilience to stress offers an eco-sustainable strategy for Smart Agricultural practices. The wider application of both natural and synthetic VOCs in most agricultural systems has focused on controlling insect pests by the VOCs acting as herbivore repellents or as attractants of their natural enemies, or on combining volatiles and pheromones for tailored herbivore trapping. The role of plant volatiles in intra- and inter-specific communication, the chemical diversity of VOCs, and their mode of action have been relatively well-documented. Studies have indicated the ecological and functional relevance of VOC-mediated communication, including their priming effect in non-stressed neighbouring plants for augmented defence responses following future stress challenges. Interestingly, most studies to date have mainly focused on biotic stress, with only a minority addressing several abiotic stresses. Moreover, information on the relevant signalling pathways and mechanisms involved in volatile-mediated plant–plant interactions remains nascent and has been sparsely studied in stress physiology studies. 

Efforts directed towards metabolite VOC profiling of plant volatile blends emitted under various environmental stresses, the identification of the specific VOC receptors, and TFs and other regulatory proteins involved in the signalling pathways are still required. Future endeavours will benefit greatly from technological advances in the fields of plant physiology, metabolomics, transcriptomics, and bioinformatics that have emerged over the past decade. Indeed, the integration of physiological, metabolomic, and transcriptomic analyses in volatile-mediated signalling and defence priming studies will contribute to achieving a more holistic understanding of VOC-induced defence responses in plants under both biotic and abiotic stresses. As most studies have been carried out under controlled environments such as glasshouses and growth chambers, it would be worthwhile to integrate such studies with those that consider more realistic environments in which plants grow. Plants in their natural environments are part of a community of constantly interacting hetero- and conspecifics and are prone to multiple stresses occurring simultaneously. Investigations into the responses by neighbouring plants to the resultant VOC emissions will provide more conclusive results regarding the feasibility of implementing VOC-mediated priming not only against biotic stresses, but also against various abiotic stresses in agricultural systems. If plants are able to manipulate responses in their neighbours against biotic stresses via VOCs, it is plausible that they could also “warn” them against impending abiotic stresses. The potential of integrating plant–plant VOC-mediated defence priming into existing plant protection strategies against a myriad of abiotic stresses appears convincing and would potentially assist in overcoming major yield-limiting factors that contribute to over 70% of crop yield losses worldwide. As indicated in this review, however, additional studies, are still required in the face of growing pressures on ecosystems and the need to drastically improve the sustainability of agricultural production.

## Figures and Tables

**Table 1:** Volatile-mediated priming in plant–plant communication under biotic and abiotic stress conditions.

Plant System	Stress Stimulus	Emitter VOCs Identified	Priming Effect on Receiver	References
Lima bean (*Phaseolus lunatus)*	Herbivory: Red spidermite (*Tetranychus urticae*)	β-Ocimene *(E*)-4,8-Dimethylnona-1,3,7-triene (DMNT) 4,8,12-Trimethyltrideca-1,3,7,11-tetraene (TMTT) Linalool (*E*)-2-Hexenal	Increase in ethylene, JA Upregulation of ethylene biosynthesis genes *S*-adenosylmethionine (SAM), 1-aminocyclopropane-1-carboxylic acid oxidase	[7]
Maize (*Zea mays)*	Mechanical damage Herbivory: Beet armyworm (*Spodoptera exigua)*	GLVs Terpenoids	Rapid JA production on post challenge with *S. exigua* regurgitant and mechanical damage	[8]
Maize (*Zea mays)*	Herbivory: Cotton leaf worm *(Spodoptera littoralis)*	GLVs Linalool (*E*)-4,8-Dimethyl-1,3-7-nonatriene (DMNT) Phenethyl acetate Indole Geranyl acetate (*E*)-β-Caryophyllene (*E*)-α-Bergamotene (*E*)*-*β-Farnesene β-Sesquiphellandrene	Direct response: reduction in caterpillar development and feeding on plant Indirect response: enhanced aromatic and terpenoid emissions resulting in increased attraction of parasitic wasp, *Cotesia marginiventris* Increase in JA-inducible genes	[9]
Tomato (*Solanum lycopersicum)*	Herbivory: Tobacco cutworm *(Spodoptera litura)*	*(Z*)-3-Hexen-1-ol	Absorbed (*Z*)-3-hexen-1-ol processed to its glycoside, (*Z*)-3-hexenyl vicianoside, reducing development and longevity of the cutworms	[12]
Sagebrush (*Artemisia tridentata)* and wild tobacco (*Nicotiana. attenuata)*	Mechanical damage (clipping)	*(Z)-*3-Hexenal *(Z)*-3-Hexen*-*1*-*ol (*Z)*-3-Hexanyl acetate Methacrolein (3*R*,7*S*)-Methyl jasmonate	Enhanced herbivore defence Increased proteinase inhibitor (PI) Increased polyphenol oxidase (PPO)	[5]
Sagebrush (*Artemisia tridentata*) and wild tobacco (*N. attenuata*)	Mechanical damage	β-Pinene 1,8-Cineole (*E*)-Ocimene *p-*Cymene Camphor Linalool β-Phellandrene Artemisole (*Z)-*3-Hexenal (*Z*)*-3-*Hexen*-*1*-*ol (*Z*)*-3-*Hexanyl acetate	Enhanced defence against herbivore *Manduca sexta* Increase in trypsin PI Upregulation of threonine deaminase and pathogen-inducible α-dioxygenase (PIOX_NICAT) Downregulation of tobacco NtPII10 photosystem II protein and ribulose 1,5-bisphosphate carboxylase subunit	[31]
*Arabidopsis thaliana*	Salinity	VOCs unknown	Increased salt tolerance, independent of ABA and salinity stress-signalling pathways	[11]
Basil (*Ocimum basilicum*)	Salinity	Bornyl acetate Eugenol *cis*-α-Bergamotene Methyl eugenol 1,8-Cineole	Higher reproductive success due to early flowering and senescence and higher seed yield VOC fingerprint that overlapped, for most compounds, with that of emitters	[28]
Broad bean (*Vicia faba)*	Salinity	VOCs unknown	Increased salt tolerance Increased stomatal conductance, photosynthetic rate, relative growth	[13]
Tea (*Camellia sinensis*)	Cold	Geraniol Linalool Nerolidol MeSA	Higher Fv/Fm values in receivers Increased cold tolerance Upregulation of dehydration-responsive elements (DRE)- binding proteins	[29]

**Table 2:** Practical technologies used in plant volatile metabolite profiling.

Methodology	Real Time Yes/No	Portability Yes/No	References
Static headspace solid-phase microextraction device (SHS-SPME) with conventional gas chromatography-mass spectrometry (GC-MS)	No	No	[239,240,241,242,243]
Dynamic purge- and -trap headspace (P&T-HS) with conventional GC-MS	No	No	[239,242,243]
Polydimethylsiloxane (PDMS) silicone tubing coupled with thermal desorption GC-MS	No	No	[252]
Portable gas chromatograph (GC) with photoionisation detector (GC-PID)	No	Yes	[249]
Micro gas chromatograph (GC) with photoionisation detector (μGC–PID)	No	Yes	[250]
GC coupled to an ion mobility spectrometer (IMS), e.g., FlavourSpec	No	Yes	[243,251]
Direct analysis in real time (DART) mass spectrometry	Yes	No	[248]
Proton-transfer reaction-mass spectrometry (PTR-MS)	Yes	No	[253]
Proton-transfer-reaction time-of-flight mass spectrometry (PTR-TOF-MS)	Yes	No	[254]

## Data Availability

Not applicable.
